# Leishmanicidal Metabolites from *Cochliobolus* sp., an Endophytic Fungus Isolated from *Piptadenia adiantoides* (Fabaceae)

**DOI:** 10.1371/journal.pntd.0000348

**Published:** 2008-12-16

**Authors:** Fernanda Fraga Campos, Luiz Henrique Rosa, Betania Barros Cota, Rachel Basques Caligiorne, Ana Lúcia Teles Rabello, Tânia Maria Almeida Alves, Carlos Augusto Rosa, Carlos Leomar Zani

**Affiliations:** 1 Laboratório de Química de Produtos Naturais, Centro de Pesquisas René Rachou, Fundação Oswaldo Cruz, Belo Horizonte, Minas Gerais, Brazil; 2 Departamento de Microbiologia, Instituto de Ciências Biológicas, Universidade Federal de Minas Gerais, Belo Horizonte, Minas Gerais, Brazil; 3 Laboratório de Microbiologia, Instituto de Ciências Exatas e Biológicas, Universidade Federal de Ouro Preto, Ouro Preto, Minas Gerais, Brazil; 4 Laboratório de Pesquisas Clínicas, Centro de Pesquisas René Rachou, Fundação Oswaldo Cruz, Belo Horizonte, Minas Gerais, Brazil; Yale University School of Medicine, United States of America

## Abstract

Protozoan parasites belonging to genera *Leishmania* and *Trypanosoma* are the etiological agents of severe neglected tropical diseases (NTDs) that cause enormous social and economic impact in many countries of tropical and sub-tropical areas of the world. In our screening program for new drug leads from natural sources, we found that the crude extract of the endophytic fungus *Cochliobolus* sp. (UFMGCB-555) could kill 90% of the amastigote-like forms of *Leishmania amazonensis* and inhibit by 100% Ellman's reagent reduction in the trypanothione reductase (TryR) assay, when tested at 20 µg mL^−1^. UFMGCB-555 was isolated from the plant *Piptadenia adiantoides* J.F. Macbr (Fabaceae) and identified based on the sequence of the internally transcribed spacer (ITS) regions of its ribosomal DNA. The chromatographic fractionation of the extract was guided by the TryR assay and resulted in the isolation of cochlioquinone A and isocochlioquinone A. Both compounds were active in the assay with *L. amazonensis*, disclosing EC_50_ values (effective concentrations required to kill 50% of the parasite) of 1.7 µM (95% confidence interval = 1.6 to 1.9 µM) and 4.1 µM (95% confidence interval = 3.6 to 4.7 µM), respectively. These compounds were not active against three human cancer cell lines (MCF-7, TK-10, and UACC-62), indicating some degree of selectivity towards the parasites. These results suggest that cochlioquinones are attractive lead compounds that deserve further investigation aiming at developing new drugs to treat leishmaniasis. The findings also reinforce the role of endophytic fungi as an important source of compounds with potential to enter the pipeline for drug development against NTDs.

## Author Summary

Protozoans belonging to genera *Leishmania* and *Trypanosoma* are single-cell organisms that can infect humans and cause disfiguring lesions and debilitating or fatal diseases, with enormous social and economic impact in many tropical and sub-tropical areas of the world. The drugs currently available to treat the different forms of leishmaniasis and trypanosomiasis were introduced many decades ago and have significant drawbacks, especially in terms of efficacy, length of treatment, route of administration, toxicity, and cost. In our screening program for new natural products with leishmanicidal activity, we found that the crude extract of a fungus living within the plant *Piptadenia adiantoides* could kill 90% of the amastigote-like forms of *Leishmania amazonensis*. The bioassay-guided fractionation of the extract resulted in the isolation of cochlioquinone A and isocochlioquinone A, which showed EC_50_ values (effective concentrations required to kill 50% of the parasite) of 1.7 µM and 4.1 µM, respectively. These compounds were not active against three human cancer cell lines (MCF-7/mammary, TK-10/renal, and UACC-62/melanoma), indicating some degree of selectivity towards the parasites. Our results suggest that cochlioquinones may serve as starting points for developing new drugs to treat leishmaniasis and reinforce the role of endophytic fungi as an important source of natural products with relevant biological activities.

## Introduction

Protozoan parasites belonging to the genera *Leishmania* and *Trypanosoma* (order Kinetoplastida, family Trypanosomatidae) occurs in the tropical and sub-tropical areas of the world, where they cause severe diseases with huge medical, social, and economic impact to millions of people [1]. All diseases caused by these parasites are among the Neglected Tropical Diseases (NTDs) listed by the World Health Organization [1]. Different species of *Leishmania* affects over 12 million people and puts over 350 million people at risk in 88 countries; *Trypanosoma cruzi* infects approximately 8 million and puts 100 million at risk in Central and South America, and *T. brucei* infects 60 million people in 36 sub-Saharan African countries [2]. The drugs currently available to treat the different forms of leishmaniasis and trypanosomiasis were introduced many decades ago and have significant drawbacks, especially in terms of efficacy, length of treatment, route of administration, toxicity, and cost [2]. To complicate the situation, there is no new drug being developed by the major pharmaceutical industries for these diseases [3].

It is well known that plant-associated microorganisms produce a variety of metabolites with novel structures and interesting biological activities [4],[5],[6],[7]. Indeed, some medicinal properties and biological activities initially attributed to plant species were found latter to be due to the secondary metabolites produced by their endophytic microorganisms [8]. With the aim to discover new drug leads for some NTDs, we have been bioprospecting the Brazilian flora and mycota using bioassays which includes the protozoan parasite *Leishmania amazonensis* and the enzyme trypanothione reductase (TryR). This flavoenzyme is part of a complex enzymatic system present in protozoans of the order Kinetoplastida that help them to survive under oxidative stress [9]. More important, TryR was shown to be essential for the growth and survival of these parasites, and was validated as a drug target for the discovery and design of new leishmanicidal and trypanocidal drugs [9],[10],[11],[12]. In our bioprospecting program, we have prepared hundreds of extracts of endophytic fungi isolated from plants growing in different Brazilian biomes (unpublished results). The isolate UFMGCB-555, obtained from *Piptadenia adiantoides* J.F. Macbr (Fabaceae), showed strong activity in the assay with TryR and *L. amazonensis* and was chosen for investigation aiming at its bioactive components. In this report we describe the molecular taxonomic identification of UFMGCB-555 and the isolation and identification of two leishmanicidal compounds from its extract.

## Methods

### Cultivation of the endophytic fungus UFMGCB-555

The endophytic fungus was isolated from *Piptadenia adiantoides* J.F. Macbr (Fabaceae) and deposited at Coleção de Microrganismos e Células da Universidade Federal de Minas Gerais under the code UFMGCB-555. The fungus was grown in potato dextrose agar (PDA) medium for 5 days at 28±2°C. Five millimeter diameter plugs of this culture were then inoculated at the center of 160 Petri dishes (90 mm diameter), each containing 20 mL of malt extract agar (MEA) medium (1% glucose, 1% malt extract, 0.1% peptone, and 1.5% agar). The plates were incubated at 28±2°C for 14 days. After this period, a small aliquot of the biomass was used for extraction of DNA and the remaining material extracted with ethyl acetate for the isolation of the fungal secondary metabolites. Five plates which were not inoculated with the fungus were subjected to the same protocol to serve as control of the culture medium.

### Molecular identification of the fungus UFMGCB-555

The DNA was extracted according to the protocol described by de Hoog [13]. The ribosomal DNA internal transcribed spacer domains (rDNA-ITS) were amplified using the primers ITS1 (5′-TCCGTAGG-TGAACCTGCGG-3′) and ITS4 (5′-TCCTCCGCTTATTGATATGC-3′), as previously described [14]. Five sequences were generated using MEGABACE (Amersham Biosciences, USA) which were used to feed PHRED-PHRAP software [15] in order to find the consensus sequence. The sequence thus obtained was compared with those deposited in the GenBank using the software BLASTn [16] to identify the fungus to the genus level. The sequence was deposited in the GenBank (accession number EU684269). Phylogenetic relationships were calculated using the version 4.0 of the software MEGA [17]. The phylogenetic tree was constructed using the neighbor joining algorithm with bootstrap values calculated from 1000 replicate runs. The Kimura 2-parameter model [18] was used to estimate the evolutionary distances.

### Extraction of secondary metabolites from the fungal cultures

The fungus culture material from 160 Petri dishes (approximately 3 liters) was transferred to a six-liter Erlenmeyer containing 3.5 L of ethyl acetate and left in contact for 48 at room temperature. After decantation, the organic phase was filtered and the solvent evaporated under vacuum in a rotary-evaporator at 45°C. Residual solvent was eliminated in a vacuum centrifuge at 40°C, and the crude extract thus obtained was stored at −40°C until use. A similar extraction procedure was carried out using the five plates containing medium only to generate a control extract of the medium.

### Chromatographic fractionation of the extract

The initial fractionation step involved high-speed counter-current chromatography (HSCCC), using a Pharma-Tech chromatograph model CCC-1000 equipped with three multilayer coils totaling a volume of 850 mL. With the rotor stopped, the coils were filled with the lower aqueous phase of the biphasic mixture of hexane-ethyl acetate-methanol-water (1.2∶0.8∶1∶1). The coils were then rotated at 1000 r.p.m. and the upper phase was pumped at a flow-rate of 5 mL min^−1^ in tail-to-head direction until hydrodynamic equilibrium was reached, that is, until only the mobile phase was flowing out of the column. Part of the extract (800 mg) was then dissolved in 10 mL of equal parts of the upper and lower phases of the biphasic solvent mixture and this mixture injected into the column. A total of 158 fractions of 15 mL each was collected. They were pooled into 40 groups based on their similarity, as assessed by thin-layer chromatography (TLC) on silica gel plates (Merck). Group 10 was fractionated on reversed-phase high performance liquid chromatography (HPLC) using a semi preparative column (250×20 mm) filled with RP-18 (octadecyl) silica gel with 5 µm average particle size. The separation was run using a gradient of methanol-water from 70 to 100% in 50 min, at a flow rate of 10 mL min^−1^. The column effluent was monitored with a UV detector set at a wavelength of 220 nm. Two pure compounds, **1** (14 mg) and **2** (2.4 mg) were isolated.

### Spectral data of the isolated compounds

Proton (^1^H) and carbon (^13^C) nuclear magnetic resonance (NMR) spectra (Figure S1), Distortionless Enhancement by Polarization Transfer (DEPT), Heteronuclear Multiple-Quantum Coherence (HMQC), and Heteronuclear Multiple Bond Correlation (HMBC) experiments were run on a Bruker DRX 400 spectrometer using the pulse programs provided by the manufacturer. The substances were dissolved in perdeuterated solvents containing 0.1% tetramethylsilane as the internal chemical shift standard. The data obtained from these spectra are summarized in the Tables 2–4. Mass spectra (MS) were acquired on a Thermo Finnigan LCQ-Advantage spectrometer equipped with an electrospray ion (ESI) source. Solutions of the compounds at 200 µg mL^−1^ in MeOH-H_2_O (1∶1) were infused at 25 µL min^−1^, and the positive and negative mass spectra acquired with a *m/z* range between 50 and 1000 daltons. The cone voltages were optimized for positive and negative ion analysis in the range between 25 and 50 V. The capillary voltages were set at 4.5 kV in positive ion mode and −3.1 kV in negative ion mode. In the MS/MS experiments, the parent ion isolation width was 3.8 daltons and the normalized collision energy was set at 30% for both compounds **1**. Fifty scans from 150 to 600 daltons were collected to generate the averaged spectra.

### Assays with recombinant trypanothione reductase from *Trypanosoma cruzi* (TryR)

The TryR microtitre plate assay procedure based on in situ Ellman's-reagent-mediated regeneration of trypanothione, described by Hamilton et al. [19], was used during the screening and the bioassay-guided fractionation protocols. The assay was performed in 96-well plates (Costar 9017, Corning, USA) using Hepes buffer (40 mM, pH 7.5) with 1 mM EDTA. Each assay well (250 µL) contained enzyme (1 mU), trypanothione (1 µM) and NADPH (200 µM). The extracts, fractions and pure compounds were added to the above mixture and incubated at 30°C during 30 min. After this period, Ellman's reagent [5,5′-dithiobis(2-nitrobenzoic acid) –DTNB] was added (70 µM) and the absorbance (Abs) measured at the wavelength of 412 nm in the kinetic mode during the time (t) of 10 minutes at every 10 seconds. The slope of the curve ΔAbs/Δt is proportional to DTNB reduction which, in absence of competing reactions, is proportional to the enzyme activity. The inhibition of the coupled system was calculated as the ratio between slope ΔAbs/Δt) of the experimental wells and that of the controls without drug, that is, percent inhibition = [1−slope_exp_/slope_contr_]×100.

The classical assay based on the measurement of NADPH consumption was performed in 1 mL cuvettes (1 cm light path length) at 27°C using a Beckman DU spectrophotometer. Each cuvette contained 500 µL Hepes buffer (40 mM, pH 7.5),1 mM EDTA, 4 mU enzyme, 200 µM NADPH, 50 µM cochlioquinone A, and 100 µM trypanothione. The enzyme, NADPH and the compound were pre-incubated for 5 minutes at 27°C. The absorbance measurement at the wavelength of 340 nm was started in the kinetic mode (1 reading per second) for about 30 seconds. The sample compartment cover was opened during 3 to 4 seconds, just enough for the quick addition of the substrate. After closing, the absorbance measurement was continued for another 30 seconds. The initial reaction velocity (v_0_) was calculated by the instrument software using the first 5–20 data points after the substrate addition. These points should fit a strait line with R^2^ (squared correlation coefficient) greater than 0.99. To estimate the effect of the compound on enzyme activity, the v_0_ values obtained in the experiments with and without cochlioquinone A were compared. Each experiment was repeated three times.

### Assays with human cancer cell lines

The effect of the extract and isolated compounds on the survival and growth of the human cancer cell lines UACC-62 (melanoma), MCF-7 (breast), and TK-10 (renal), was determined using a colorimetric method developed at the National Cancer Institute-USA [20],[21]. Briefly, the cells were inoculated in 96-well plates and incubated at 37°C for 24 h in 5% CO_2_ atmosphere. The solutions of the test samples were added to the culture wells to attain the desired concentrations, and the plates incubated for further 48 h. Trichloroacetic acid was added to each well to precipitate the proteins, which were stained with sulforhodamine B. After washing out the unbound dye, the stained protein was dissolved with 10 mM Tris, and the absorbance measured at the wavelength of 515 nm. Results were calculated using the absorbance measured in the test-wells (T) in comparison with that of the control wells corresponding to the initial cell inoculum (T_i_) and cells grown for 48 h without drug (T_f_), using the formula: [(T−T_i_)/(T_f_−T_i_)]×100. This formula allows the quantification of both growth inhibition (values between zero and 100) and cell death (values smaller than zero). Each sample was tested in duplicate in two independent experiments.

### Assay with amastigote-like forms of *Leishmania (Leishmania) amazonensis*

Promastigotes of *L. amazonensis* (strain IFLA/BR/196/PH-8) were obtained from lesions of experimentally infected hamsters. The parasites were incubated for 9 days at 26°C in Schneider's medium buffered at pH 7.2. The promastigotes were then stimulated to differentiate into amastigote-like forms by rising the incubation temperature to 32°C and lowering the pH of the medium to 6.0. After 7 days under these conditions 90% of the parasites were in the amastigote-like forms. The parasite concentration was adjusted to 1×10^8^ cells mL^−1^, and 90 µL added to each well of 96-well plates, followed by 10 µL of the solutions containing the samples and control drug (0.2 µg mL^−1^ Amphotericin B - Fungisone Bristol-Myers Squibb, Brazil). The plates were incubated at 32°C for 72 h and the number of parasites estimated using the MTT (methyl thiazolyl tetrazolium)-based colorimetric assay [22]. The results were calculated from the measured absorbancies using the formula [1−(Abs_exp_/Abs_contr_)]×100, which express the percentage of parasite death in relation to the controls without drug. All samples were tested in duplicate and the experiments repeated at least once. Experiments to determine the dose response curves and the EC_50_ (effective concentration to kill 50% of the parasites) were run as above, using 1∶2 serial dilutions of the test compounds to reach the appropriate concentrations. The experiments were run in duplicate and repeated at least once.

### Assays with microorganisms

The antimicrobial activity of the samples was evaluated using the following microorganisms: *Candida albicans* ATCC18804, *C. krusei* ATCC2159, *Staphylococcus aureus* ATCC12600, *Escherichia coli* ATCC25922 and *Cryptococcus neoformans* ATCC32608. The yeasts were grown on agar Sabouraud (Difco) at 28°C for 24 h, and their inocula were adjusted in saline solution to Mac Farland optical density scale 1 [23] before seeding into plates containing agar Sabouraud. The bacteria were grown in Brain Heart Infusion (BHI, Difco) in 6-mL tubes, and their concentration adjusted to 10^3^ to 10^4^ cells mL^−1^ before inoculation into plates containing agar BHI. In each plate, five clean filter-paper disks with 6 mm diameter were placed equidistant from each other on the surface of the medium. Solutions of the samples at 10 mg mL^−1^ were prepared in 1% aqueous dimethyl sulfoxide (1% aq. DMSO). Five microliters of these solutions, corresponding to 50 µg of the sample, were applied to the paper disks, and the plates incubated at 37°C for a period of 24 to 48 h. Aqueous solutions of amphotericin B and chloramphenicol at 10 mg mL^-1^ were used as positive controls for yeasts and bacteria, respectively. Solvent control consisted of 1% aq. DMSO. The sample was considered active if it caused a growth inhibition halo around the disk to which it was applied.

### Statistical analysis

The software GraphPad Prism version 4.03 was used to calculate the EC_50_ values using the non-linear curve fitting of two ore more independent experimental datasets to a four-parameter logistic dose-response model. No constraints were applied to the curve fitting calculations.

## Results

### Molecular identification of UFMGCB-555

**Figure 1:**
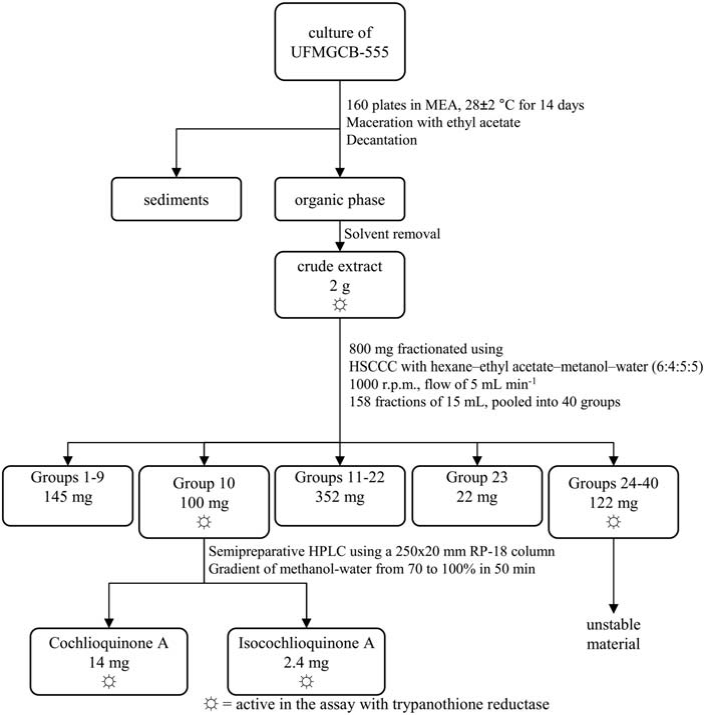
Phylogenetic tree depicting the relationships between UFMGCB-555 and related species. The tree was constructed by neighbor-joining analysis (Kimura-2 parameter) of their ITS domain sequences. Bootstrap percentage from 1000 replicates is shown. The GenBank accession numbers are indicated for each reference sequence. The ITS sequence of *Alternaria* (AY673074) was used as an outgroup.

A small aliquot (1 g) of the fungus culture was used to isolate the fungal DNA for sequencing of the ITS domains. These sequences were used for the taxonomic identification of the fungus and for the elucidation of its phylogenetic relationships (Figure 1). The fungus UFMGCB-555 was then identified to the genus level as *Cochliobolus* sp. and showed a close phylogenetic relationship with *C. melinidis* (Genebank access number AF452445), from which its consensus sequence differs by only 2.7% (12 nucleotides, 70% bootstrap value).

### Extraction of secondary metabolites

The culture material (3 liters) yielded 2 g of the crude extract, representing a yield of approximately 0.6 g L^−1^ of culture broth. This extract showed activity in different bioassays (Table 1) when tested at 20 µg mL^−1^, but was completely inactive against *C. albicans*, *C. krusei*, *S. aureus*, *E. coli* and *C. neoformans* when tested at 50 µg per disk (data not shown). The extract of the control (sterile) culture medium was not active in these assays.

**Table 1:** Biological activities of crude extract, fraction, and compounds isolated from *Cochliobolus* sp. (UFMGCB-555) tested at a single dose of 20 µg mL^−1^.

Samples	AMA^a^	TryR^b^	Cancer cell lines^c^		
			UACC-62	MCF-7	TK-10
Crude extract	87±3	99±2	62±22	67±8	80±16
Group 10	nt^d^	99±5	−	−	−
1	60±5	99±4	−	−	−
2	70±2	−	−	−	−

a Percentage of death of amastigote-like forms of *Leishmania* (*Leishmania*) *amazonensis*. Control drug: amphotericin B at 0.2 µg mL^−1^.

b Percentage inhibition of the enzyme trypanothione reductase. Clomipramine at 6 µM was used as 50% inhibition control.

c Cytocidal activity (percentage of death of human cancer cells relative to the initial inoculum). Control drugs: etoposide at 16 µg mL^−1^ and colchicine at 8 µg mL^−1^.

d not tested.

The values represent the mean±S.D. of at least 2 independent experiments.

The minus symbol (−) means inactive.

### Bioassay-guided fractionation of the extract

**Figure 2:**
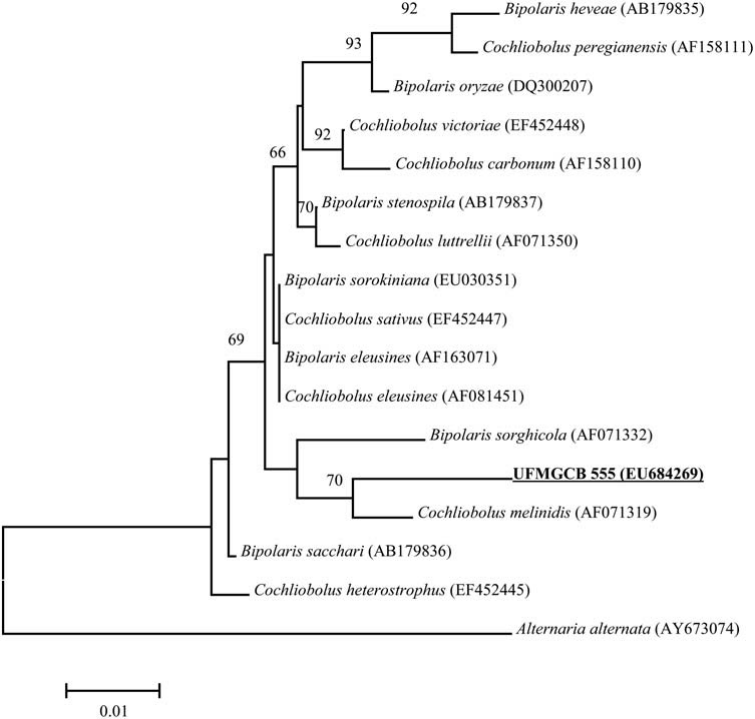
Flowchart illustrating the isolation of cochlioquinone A and isocochlioquinone A. The fractionation was guided by the bioassay with the enzyme trypanothione reductase, in which the crude extracts, fractions, and isolated compounds were tested at 20 µg mL^−1^.

Approximately 800 mg of the crude extract was subjected to counter-current chromatography in an HSCCC to afford 158 fractions, which were pooled into 40 groups (Figure 2). After testing all groups in the TryR assay, only Group 10 and Groups 24–40 showed some activity. Groups 24–40 were not studied further because they presented low masses and were constituted of complex and instable mixtures of highly polar compounds. Fraction 10 (100 mg) was further fractionated using reversed-phase HPLC to yield compounds **1** (14 mg; 1.4% w/w of the crude extract) and **2** (2.4 mg; 0.24% w/w of the crude extract).

**Figure 3:**
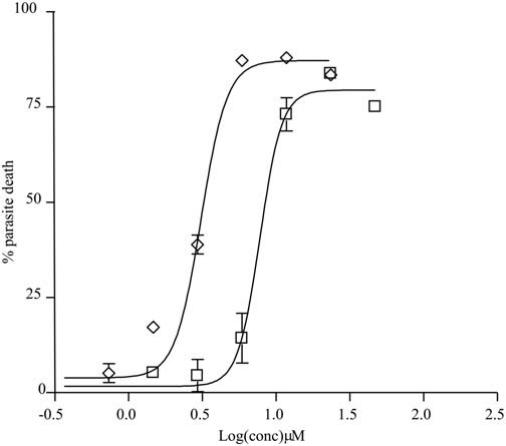
Dose response curves for 1 (diamonds) and 2 (squares) in the assay with amastigote-like forms of *L. amazonensis*. The EC_50_ (effective concentration to kill 50% of the parasites) values were found to be 1.7 µM (95% confidence interval = 1.5 to 1.9 µM) and 4.1 µM (95% confidence interval = 3.6 to 4.7 µM), respectively. The data in the plots are derived from at least two independent experiments.

Both compounds strongly inhibited the growth of amastigote-like forms of *L*. (*L*.) *amazonensis*, with EC_50_ values (effective concentration to kill 50% of the parasites) of 1.7 µM (95% confidence interval = 1.5 to 1.9 µM) and 4.1 µM (95% confidence interval = 3.6 to 4.7 µM), respectively (Figure 3). However, only **1** was active in the DTNB-coupled TryR assay. Furthermore, while the crude extract was toxic to three human cancer cell lines used in this work (MCF-7, TK-10, and UACC-62), neither Group 10 nor compounds **1** and **2** showed activity against these lineages (Table 1). The compounds were also inactive against the five pathogenic microorganisms investigated (data not shown).

### Structure elucidation of the bioactive compounds

**Figure 4:**
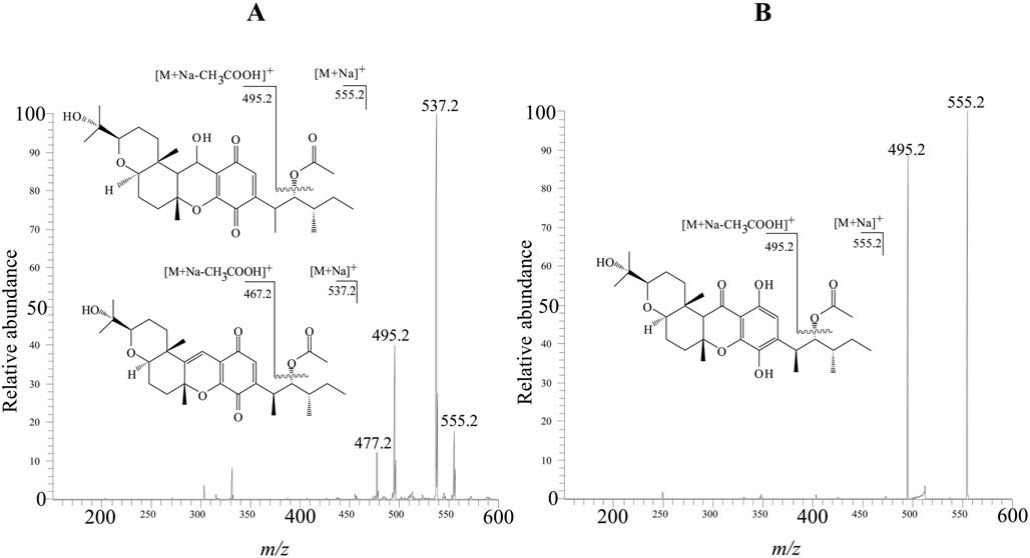
ESI-MS-MS in the positive ion mode showing the main fragments generated by cochlioquinone A (panel A) and isocochlioquinone A (panel B). Both compounds show sodiated quasi-molecular ion peaks [M+Na]^+^ at 555 daltons and the loss of acetic acid (Δ *m/z = *60 daltons) from the side chain. In addition, the hydroxyl group in the pyrane ring of cochlioquinone A is responsible for the observed loss of water (Δ *m/z* = 18 daltons).

**Figure 5:**
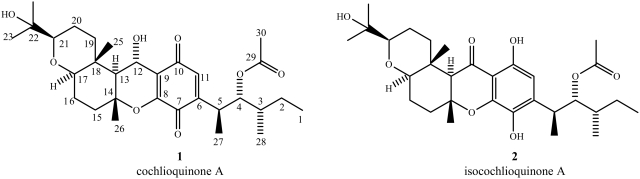
Chemical structures of compounds isolated from *Cochliobolus* sp. UFMGCB-555. The numbering in these structures follows that used in [42].

The electrospray ionization mass spectra (ESI-MS) of both compounds exhibited quasi-molecular sodiated ion peaks [M+Na]^+^ with *m/z* 555 daltons in positive ion mode, and [M-H]^−^ with *m/z* 531 daltons in negative ion mode, indicating they both have molecular weight of 532 g mol^−1^ (Figure 4). The peak integration areas in the ^1^H NMR spectra (Table 2 and Table 3, Figure S1) indicated the presence of 44 hydrogen atoms, while the ^13^C NMR spectra (Table 4) showed 30 signals for each compound. Edited DEPT sub-spectra showed signals due eight methyl groups, one belonging to an acetyl group, and five methylene carbon atoms for both compounds. Signals due to eight methyne carbon atoms, four of them oxygenated, were observed for compound **1**, while seven signals of methyne carbon atoms, three of them oxygenated, were detected for in the spectra of compound **2**. Altogether, these data is compatible with the molecular formula C_30_H_44_O_8_, with an index of hydrogen deficiency of 18, corresponding to 9 unsaturations. The analysis of the ^1^H and ^13^C spectra allow to infer that both compounds have five double bonds in their structures, with the remaining unsaturations attributed to the presence of four rings. A quinonoid ring in **1** was suggested by the presence of the signals at 181.64 and 188.86 δ, attributed to carbonyl groups, together with four signals between 134.32 and 151.40 δ in the ^13^C NMR spectrum. A phenol moiety in **2** was indicated by the presence of six signals between 107.0 and 181.5 δ in the ^13^C NMR spectrum. A signal at 198.5 δ suggested the presence of a ketone carbonyl in **2**. The connections between carbon and hydrogen atoms in the structures were established based on the analysis of the two-dimensional NMR experiments (COSY, HMQC and HMBC - Table 2 and Table 3). The spectral data of **1** and **2** are in agreement with those published in the literature for cochlioquinone A and isocochlioquinone, respectively (Figure 5).

**Table 2:** ^1^H-NMR and HSQC spectral data of **1** and comparison with literature data for cochlioquinone A.

Position	Cochlioquinone A^a^		1	
	Chemical shifts	Multiplicity and coupling constants	Chemical shifts	Multiplicity and coupling constants
1	0.88	(3H, *t, J = *7.0)	0.85	(3H, *t, J = *6.8)
2	1.35	(2H, *)	1.45	(1H, *)
			1.08	(1H, *)
3	1.60	(1H, *)	1.49	(1H, *)
4	5.01	(1H, *dd, J = *7.5, 5.0)	4.92	(1H, *dd, J = *7.7, 5.1)
5	3.22	(1H, *)	3.37	(1H, *dq, J = *7.0, 5.1)
11	6.63	(1H, *s*)	6.63	(1H, *s*)
12	4.93	(1H, *dd, J = *10.0, 1.3)	4.94	(1H, *d, J = *10.6)
13	1.72	(1H, *d, J = *10.0)	1.72	(1H, *d, J = *10.6)
15	1.91	(1H, *dt, J = *13.3, 3.8)	1.91	(1H, *dt, J = *13.2, 3.9)
	2.09	(1H, *dt, J = *13.3, 3.3)	2.08	(1H, *dt, J = *13.2, 3.5)
16	1.56	(1H, *)	1.54	(1H, *)
	1.79	(1H, *)	1.79	(1H, *)
17	3.17	(1H, *dd, J = *12.5, 3.8)	3.17	(1H, *dd, J = *11.9, 3.5)
19	1.40	(1H, *)	1.40	(1H, *)
	2.47	(1H, *m*)	2.47	(1H, *m*)
20	1.43	(1H, *)	1.43	(1H, *)
	1.65	(1H, *)	1.64	(1H, *)
21	3.25	(1H,*dd, J = *12.5, 2.5)	3.25	(1H,*dd, J = *11.9, 2.8)
23	1.17	(3H, *s*)	1.16	(3H, *s*)
24	1.18	(3H, *s*)	1.18	(3H, *s*)
25	1.01	(3H, *s*)	1.01	(3H, *s*)
26	1.32	(3H, *s*)	1.32	(3H, *s*)
27	1.14	(3H, *d, J = *6.8)	1.12	(3H, *d, J = *7.0)
28	0.87	(3H, *d, J = *7.0)	0.84	(3H, *d*, *J = *7.5)
30	1.98	(3H, *s*)	2.05	(3H, *s*)

Spectra were recorded at 400 MHz in CDCl_3_.

a data from reference [28].

*J* = coupling constant in Hz.

*: overlapping multiplet signal.

**Table 3 pntd-0000348-t003:** ^1^H-NMR and HSQC spectral data of **2** and comparison with literature data for isocochlioquinone A.

Position	Isocochlioquinone A^a^		2	
	Chemical shifts	Multiplicities and coupling constants	Chemical shifts	Multiplicities and coupling constants
1	0.89	(3H, *t, J = *7.0)	0.83	(3H, *t, J = *7.4)
2	1.25	(2H, *)	1.45	(1H, *)
			1.08	(1H, *)
3	1.63	(1H, *)	1.56	(1H, *)
4	5.18	(1H, *dd, J = *7.5, 5.0)	5.04	(1H, *dd, J = *7.6, 5.3)
5	3.46	(1H, qnt, *J = *7.0)	3.37	(1H, *dq, J = *7.0, 5.3)
11	6.40	(1H, *s*)	6.52	(1H, *s*)
13	2.77	(1H, *s*)	2.78	(1H, *s*)
15	2.06	(1H, *)	2.08	(2H, *)
	2.09	(1H, *dt, J = *13.3, 3.3)		
16	1.60	(1H, *)	1.66	(1H, *)
	1.80	(1H, *)	1.81	(1H, *ddd*, *J = *13.4, 7.33, 3.8)
17	3.16	(1H, *dd, J = *12.0, 3.8)	3.15	(1H, *dd, J = *11.9, 3.8)
19	1.30	(2H, *)	1.29	(1H, *m*)
	2.75	(1H, *)	2.75	(1H, *m*)
20	1.60	(1H, *)	1.46	(1H, *)
			1.74	(1H, *)
21	3.27	(1H,*dd, J = *12.5, 2.5)	3.26	(1H,*dd, J = *12.0, 2.9)
23	1.19	(3H, *s*)	1.19	(3H, *s*)
24	1.19	(3H, *s*)	1.19	(3H, *s*)
25	1.13	(3H, *s*)	1.01	(3H, *s*)
26	1.46	(3H, *s*)	1.48	(3H, *s*)
27	1.20	(3H, *d, J = *7.0)	1.19	(3H, *d, J = *7.0)
28	0.88	(3H, *d, J = *6.8)	0.86	3H, *d*, *J = *6.6
30	1.93	(3H, *s*)	2.05	(3H, *s*)

Spectra were recorded at 400 MHz in CDCl_3_.

a data from reference [28].

*J* = coupling constants in Hz.

*: overlapping multiplet signal.

**Table 4:** ^13^C-NMR and DEPT spectral data of compounds **1** and **2** and comparison with literature data for Cochlioquinone A and Isocochlioquinone.

Position	Chemical shifts		Mult^b^	Chemical shifts		Mult^b^
	1	Cochlioquinone A^a^		2	Isocochlioquinone A^a^	
1	11.1	11.4	CH3	11.1	11.4	CH3
2	25.2	26.4	CH2	24.2	26.5	CH2
3	36.4	36.2	CH	36.4	36.2	CH
4	79.9	78.2	CH	80.6	79.0	CH
5	32.7	34.6	CH	34.4	35.5	CH
6	148.1	148.3	qC	139.7	140.2	qC
7	181.6	181.5	qC	135.0	135.3	qC
8	151.4	151.5	qC	143.8	144.0	qC
9	119.1	119.0	qC	107.0	107.0	qC
10	188.9	188.5	qC	153.2	153.2	qC
11	134.3	133.6	CH	108.2	108.2	CH
12	63.1	63.0	CH	198.3	198.5	C
13	51.7	51.8	CH	60.4	60.5	CH
14	83.2	83.0	qC	83.5	83.3	qC
15	37.5	37.5	CH2	37.6	37.6	CH2
16	24.0	25.2	CH2	25.0	25.0	CH2
17	83.8	83.8	CH	83.6	83.6	CH
18	36.7	36.7	qC	35.5	35.6	qC
19	38.6	38.5	CH2	37.4	37.3	CH2
20	21.5	21.5	CH2	21.3	21.3	CH2
21	85.1	85.1	CH	85.4	85.5	CH
22	71.9	71.7	qC	71.9	72.0	qC
23	23.8	24.0	CH3	23.8	23.8	CH3
24	26.1	25.9	CH3	26.0	26.0	CH3
25	12.6	12.6	CH3	12.3	12.5	CH3
26	21.1	21.0	CH3	22.0	22.0	CH3
27	18.2	17.2	CH3	18.0	17.5	CH3
28	15.4	13.2	CH3	15.3	13.5	CH3
29	170.6	170.3	qC	170.8	170.5	qC
30	20.9	20.7	CH3	21.0	20.8	CH3

Spectra were recorded at 100 MHz in CDCl_3_.

a data from reference [28].

b the multiplicities were deduced from DEPT experiments; qC means quaternary carbon.

## Discussion

Several endophytic fungi were isolated from the *P. adiantoides*, a plant species selected due to the activity of its extract in a panel of assays used to screen the Brazilian flora for bioactive natural products (unpublished results). Among the fungi isolated from this plant, the isolate UFMGCB-555 showed strong activity in the assays with TryR and *L. amazonensis.* Using molecular taxonomy techniques, we were able to identify this fungus as *Cochliobolus sp.* (Pleosporaceae, Ascomycota). This genus comprises approximately 50 species occurring all over the world [24], many of which can parasitize plants and cause considerable agricultural losses [25]. Some species of *Bipolaris*, the anamorphic state of *Cochliobolus*, are also the etiologic agent of several human diseases, such as sinusitis, ocular infections, peritonitis, and meningoencephalitis [26],[27]. However, in the present work we looked for the ability of *Cochliobolus* UFMGCB-555 to produce secondary metabolites with biological or pharmaceutical potential, especially for neglected tropical diseases.

To guide the fractionation process aiming at the isolation of the active compounds of this extract, we decided to use the TryR assay developed by Hamilton et al. [19]. Besides being more economic due to low consumption of the expensive substrate trypanothione, this assay is simpler, safer and faster to perform than the assay with *L. amazonensis*. Thus, the bioassay with the parasite was used only at the end of the isolation procedure, with the pure compounds. Using this strategy we arrived at a fraction containing two major substances, one active (**1**) and the other inactive (**2**) in the TryR assay (Table 1). Both compounds were, however, active against *L. amazonensis*, showing EC_50_ values of 1.7 µM and 4.1 µM, respectively (Figure 3).

After detailed analysis of the ESI-MS and NMR spectra, and comparison of our data with those published in the literature [28], compounds **1** and **2** were identified as cochlioquinone A and isocochlioquinone, respectively (Figure 5). However, slightly different interpretation of some NMR signals, as compared with those described by Miyagawa [28], are noteworthy: a) the hydrogen at C-5 in both compounds (Table 2 and Table 3–position 5) couples with the three hydrogen atoms bound to C-27 and with the hydrogen at C-4, thus appearing as a double quartet, with coupling constants *J = * 7.0 and 5.1 Hz, and not as quintet, as described by Miyagawa; b) the two hydrogen atoms at C-2 in compound **1** (Table 2–position 2) show distinct signals centered at 1.45 δ and 1.08 δ and not one centered at 1.35 δ; c) the same is true for compound **2** (Table 3–position 2), where the corresponding signals are observed at 1.45 and 1.08 δ and not only at 1.25 δ, and finally; d) the two hydrogen atoms at C-20 in compound **2** (Table 3–position 20) resonate at 1.46 δ and 1.74 δ and not only at 1.60 δ as described by those authors.

Cochlioquinones and related compounds are known to occur in fungi from different genera, including *Cochliobolus* and *Bipolaris* [29],[30],[31], *Helminthosporium* [32], *Drechslera* [33], and *Stachybotrys* [30]. However, this is the first report on the leishmanicidal activity of compounds belonging to this class of natural products.

**Figure 6:**
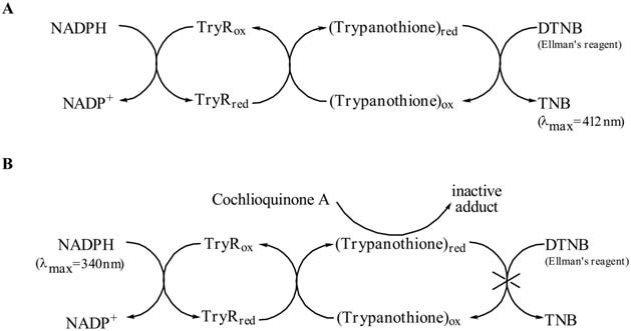
(A) Schematic view of the chemical and enzymatic reactions involved in the TryR assay resulting in the reduction of the Elmman's reagent (DTNB) to the yellow-colored 2-nitro-5-thiobenzoic acid (TNB); (B) Same as above, showing the interference of cochlioquinone A resulting in a false positive readout due to its reaction with the reduced form of trypanothione, interrupting the chemical reduction of DTNB.

It is known that quinonoid compounds with free positions in the quinone ring are susceptible to nucleophilic attack by thiol groups to form stable Michael addition products [34]. In this regard, after **1** was unequivocally identified as cochlioquinone A, its activity in the Ellman's-reagent-mediated TryR assay was questioned due to the presence of a quinonoid ring in its structure. Thus, rather than directly inhibiting the enzyme in the assay, cochlioquinone A could be capturing the reduced substrate resulting in the interruption of the chemical reduction of Ellman's reagent (Figure 6). To confirm this possibility, we performed the assay using the classical protocol based on the measurement of NADPH consumption [35] while using an excess trypanothione (100 µM) in comparison to cochlioquinone A (50 µM). Indeed, under these conditions no inhibition of the enzyme activity could be observed (data not shown).

In view of the above results, we can conclude that cochlioquinone A and isocochlioquinone A are exerting their leishmanicidal effect by hitting targets other than TryR within the parasite. A literature search disclosed the following information: a) cochlioquinone A is a competitive inhibitor of the ivermectin binding site in *Caenorhabdites elegans*, with an inhibition constant of 30 µM [36]; b) ivermectin is also active *in vivo* against different species of *Leishmania* [37],[38]; c) the mode of action of ivermectin in nematodes is related to its high affinity to glutamate-gated chloride channels, causing an increase in the permeability of the cell membrane to chloride ions [39]; d) the TDR database of potential drug targets for NDTs (http://tdrtargets.org) reveals four genes of *L. major* expressing putative chloride ions transporters (LmjF01.0180, LmjF04.1000, LmjF32.3370, and LmjF33.1060); e) at least three of these genes have orthologs in *C. elegans* (C07H4.2, R07B7.1), while LmjF01.0180 has an ortholog also in *T. cruzi* (Tc00.1047053504797.140). Based on these pieces of information it is plausible to speculate that chloride ions transporters may also serve as a target for cochlioquinone A and related compounds in *Leishmania* and *Trypanosoma*. This hypothesis needs further experimental evidences to be confirmed or refuted.

Concerning isocochlioquinone A, the literature [40] show that it can inhibit the growth of the malaria-causing protozoan parasite *Plasmodium falciparum* with IC_50_ values of 1.4 µg mL^−1^ for the K1 strain (resistant to chloroquine and pyrimethamine), and 3.3 µg mL^−1^ for the NF 54 strain (susceptible to standard antimalarials). These values are close to the EC_50_ shown against *L. amazonensis* in the present work.

Besides the significant activity against *L. amazonensis*, our data indicate that **1** and **2** present some degree of selectivity, as they were inactive in the assays with three human cancer cell lines (Table 1) and five pathogenic microorganisms (data not shown) used in this investigation. The low toxicity of **1** and **2** to mammalian cell lines reported in this work is in agreement with recently reported data showing that isocochlioquinone A has only a small effect on HeLa and KB cells [29],[41]. Another study [33] showed that when cochlioquinone A was tested *in vitro* against different kinases it showed selective activity against diacylglycerol kinase, both *in vitro* and in whole cell assay employing BW5147 T cell lymphoma lineage. Related compounds, such as cochlioquinone A1, exhibited selective toxicity towards bovine aortic endothelial cell when compared with normal and cancer cell lines [29] leading the authors to suggest that it may serve for developing new therapeutic agents for angiogenesis-related diseases.

The activity in the low micro molecular range towards *L. amazonensis* and the selectivity of **1** and **2** are reported here for the first time and justify further investigations on compounds of this class to assess their *in vitro* and *in vivo* effect on parasites of the genera *Leishmania* and *Trypanosoma*. Finally, by disclosing the leishmanicidal activity of two secondary metabolites from an endophytic fungus, the present work reinforces the role of these organisms as an important source of drug lead candidates for the development of new chemotherapeutic agents for NTDs.

## Supporting Information

Figure S1 ^1^H and ^13^C NMR spectra of the isolated compounds.
(0.09 MB PDF)
