# Correction: Leishmanicidal Metabolites from *Cochliobolus* sp., an Endophytic Fungus Isolated from *Piptadenia adiantoides* (Fabaceae)

**DOI:** 10.1371/annotation/49748d99-fe4d-4c28-b77a-306d0cf7062e

**Published:** 2009-01-02

**Authors:** Fernanda Fraga Campos, Luiz Henrique Rosa, Betania Barros Cota, Rachel Basques Caligiorne, Ana Lúcia Teles Rabello, Tânia Maria Almeida Alves, Carlos Augusto Rosa, Carlos Leomar Zani

The images for Figures 1 and 2 were incorrectly switched. The image that appears as Figure 1 should be Figure 2, and the image that appears as Figure 2 should be Figure 1. The figure legends are numbered correctly.

