# Supplementary material for: Leishmanicidal Metabolites from Cochliobolus sp., an Endophytic Fungus Isolated from Piptadenia adiantoides (Fabaceae)
Source: PLoS Negl Trop Dis. 2008 Dec 16;2(12):e348. doi: 10.1371/journal.pntd.0000348 (PMC2593781; doi:10.1371/journal.pntd.0000348)

Cochlioquinone 1H NMR

22 Sep 2008
Isolated from Cochliobolus sp

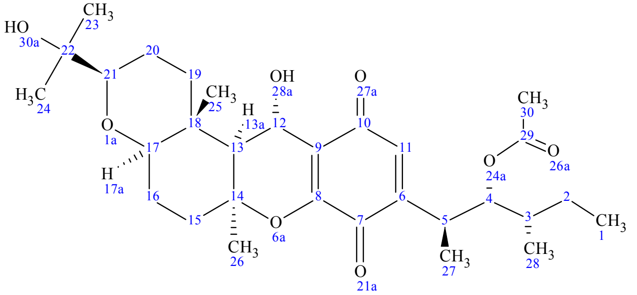

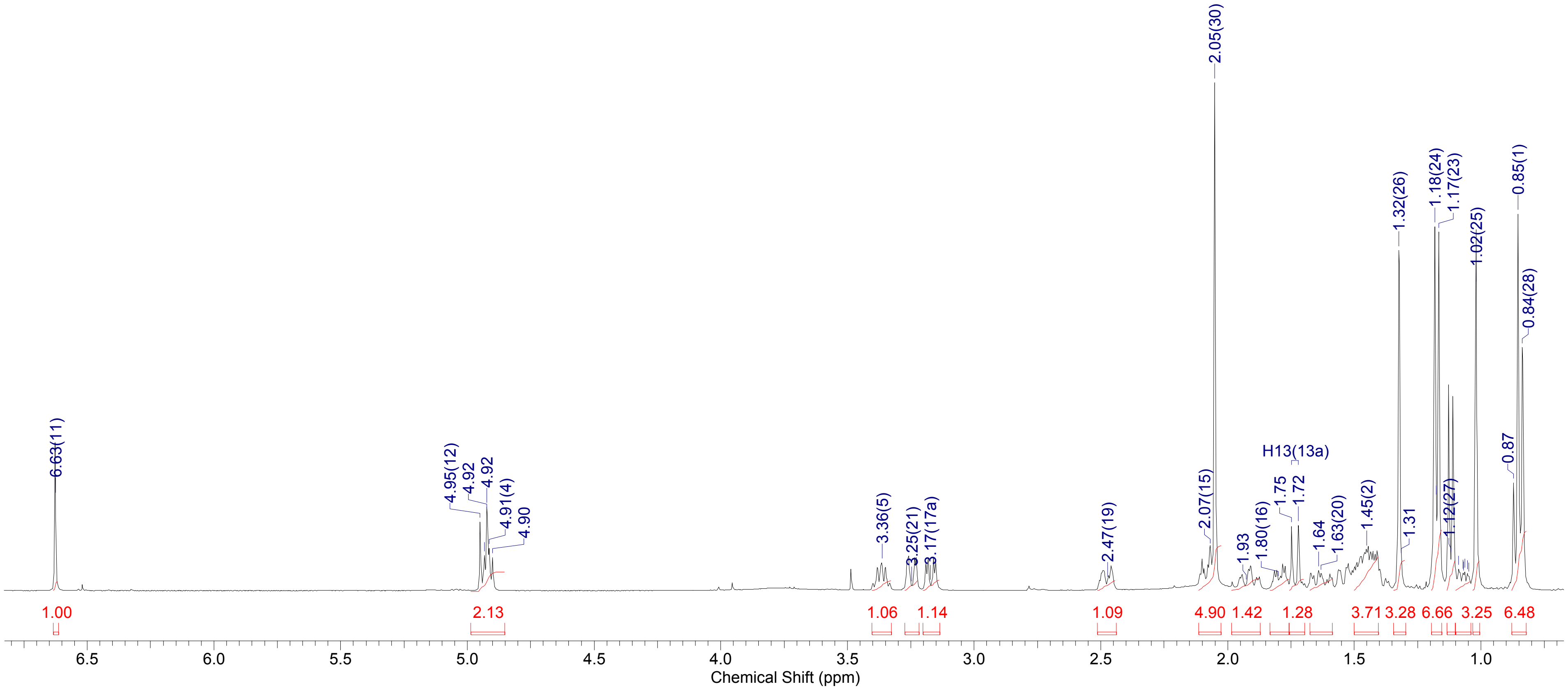

Cochlioquinone 13C NMR

Isolated from Cochliobolus sp

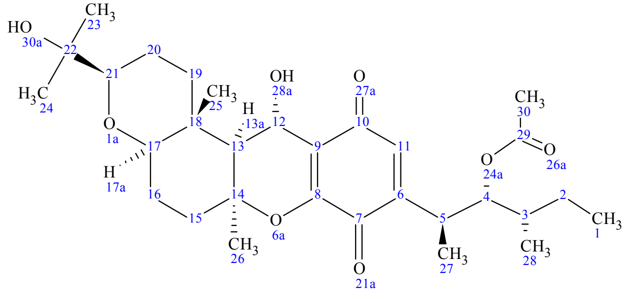

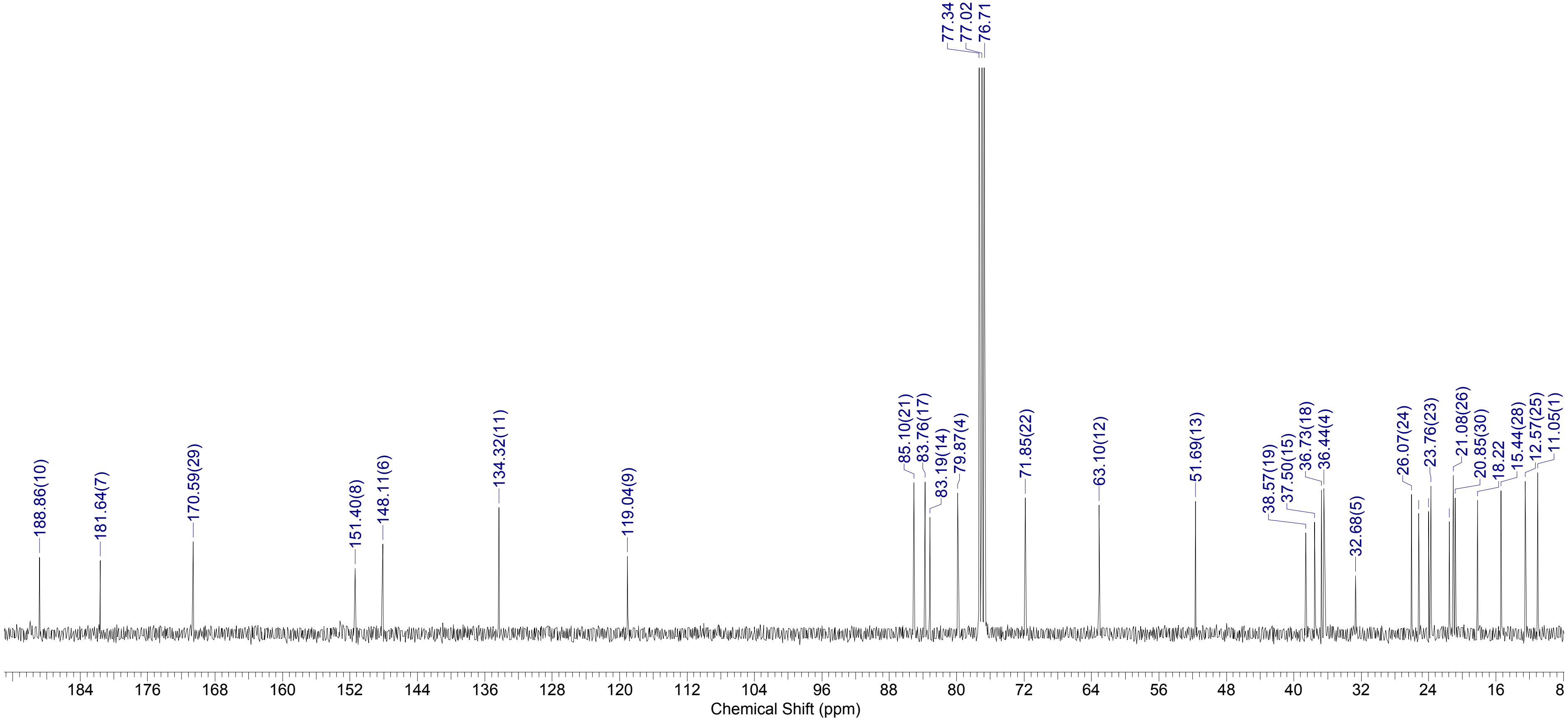

Isocochlioquinone 1H NMR

Isolated from Cochliobolus sp

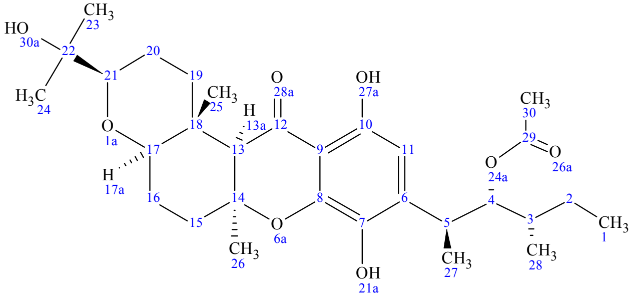

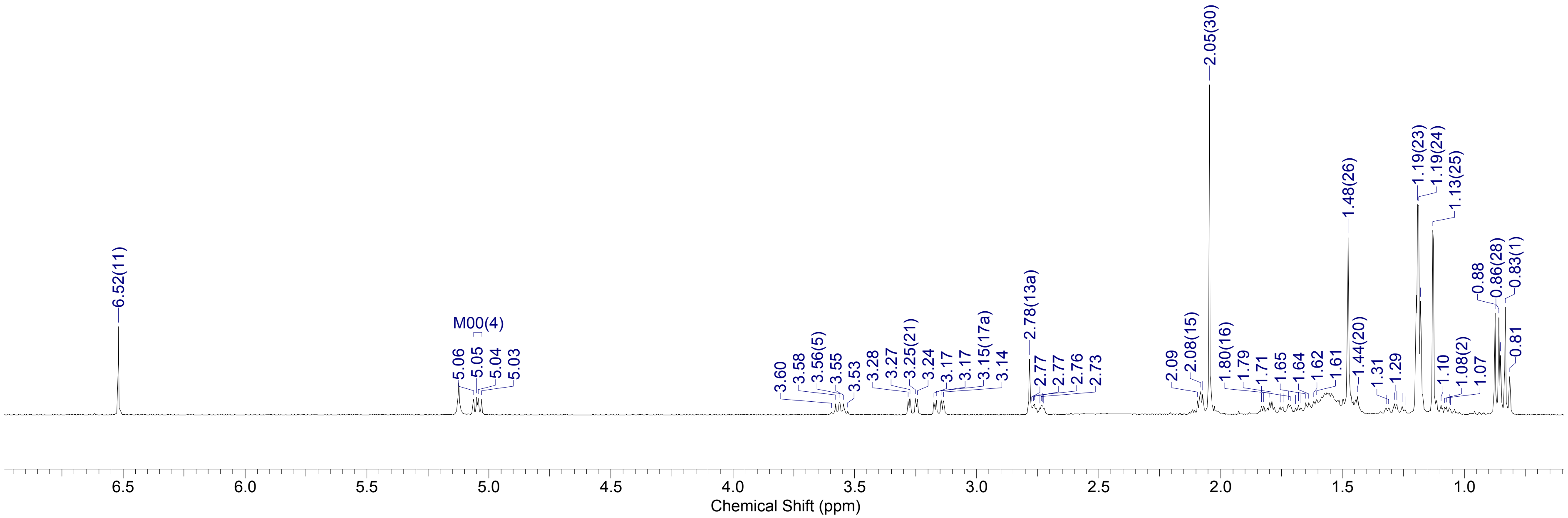

Isocochlioquinone 13C NMR

Isolated from Cochliobolus sp

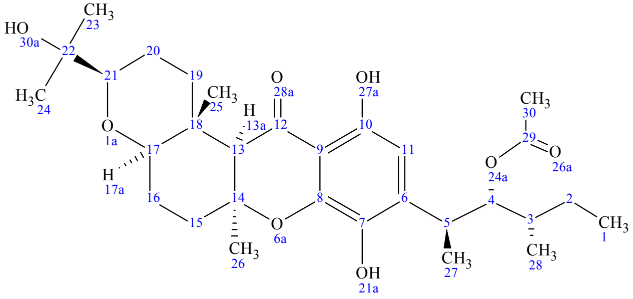

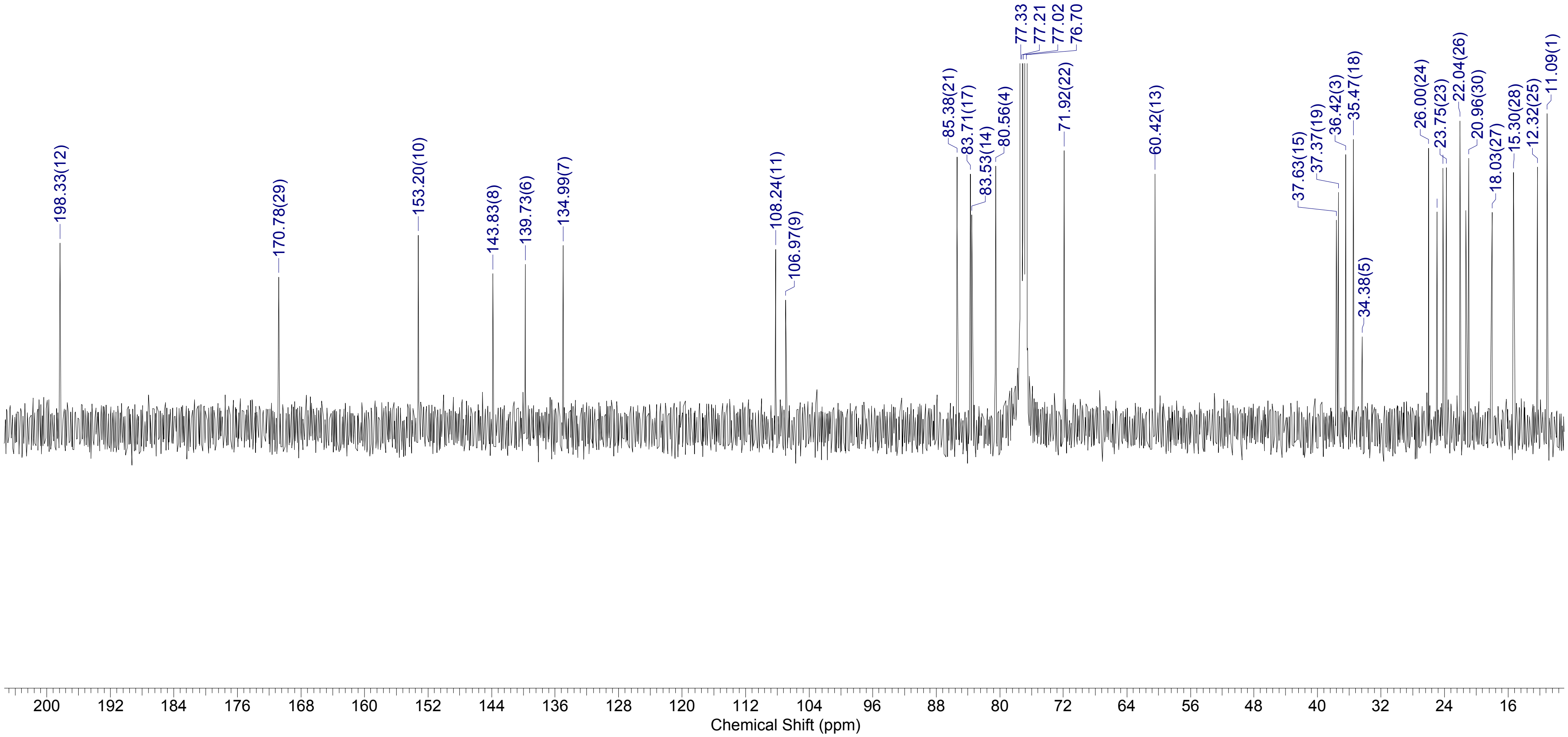

Supplement: Figure S1 — 1H and 13C NMR spectra of the isolated compounds. (0.09 MB PDF) [file pntd.0000348.s001.pdf]
